# Arithmetic Framework to Optimize Packet Forwarding among End Devices in Generic Edge Computing Environments

**DOI:** 10.3390/s22020421

**Published:** 2022-01-06

**Authors:** Pedro Juan Roig, Salvador Alcaraz, Katja Gilly, Cristina Bernad, Carlos Juiz

**Affiliations:** 1 Computer Engineering Department, Miguel Hernández University, 03202 Elche, Spain; salcaraz@umh.es (S.A.); katya@umh.es (K.G.); cbernad@umh.es (C.B.); 2 Mathematics and Computer Science Department, University of the Balearic Islands, 07022 Palma de Mallorca, Spain; cjuiz@uib.es

**Keywords:** edge computing, fog computing, formal modeling, ACP, IoT

## Abstract

Multi-access edge computing implementations are ever increasing in both the number of deployments and the areas of application. In this context, the easiness in the operations of packet forwarding between two end devices being part of a particular edge computing infrastructure may allow for a more efficient performance. In this paper, an arithmetic framework based in a layered approach has been proposed in order to optimize the packet forwarding actions, such as routing and switching, in generic edge computing environments by taking advantage of the properties of integer division and modular arithmetic, thus simplifying the search of the proper next hop to reach the desired destination into simple arithmetic operations, as opposed to having to look into the routing or switching tables. In this sense, the different type of communications within a generic edge computing environment are first studied, and afterwards, three diverse case scenarios have been described according to the arithmetic framework proposed, where all of them have been further verified by using arithmetic means with the help of applying theorems, as well as algebraic means, with the help of searching for behavioral equivalences.

## 1. Introduction

Multi-Access Edge Computing (MEC) environments are growing by the day due to the need of more computing power near the end users [1]. Some years ago, artificial intelligence (AI) techniques, such as Convolutional Neural Networks (CNN), were only available in cloud facilities [2], due to their high requirements of computing resources and power, which were only available through Wide Area Network (WAN) links, those having constraint bandwidth and large round trip times. However, recent advances in the data science (DS) paradigm has brought up a new generation of smaller AI-powered resources, which may be embedded into smaller facilities, such as those being implemented into the fog [3] or into the edge [4], which are reachable through Local Area Network (LAN) links, those having greater bandwidth and shorter latency rates.

Specifically, those new AI-powered may improve performance in many fields [5], and may allow Internet of Things (IoT) devices to take advantage of them by means of Local Area Network (LAN) links, those having high bandwidth and short round trip times [6]. This situation may facilitate the increase in IoT environments [7], which may induce the need of optimizing their designs [8], and in this regard, it might be useful to obtain a generic regular scheme in order to optimize the resources being put in place when undertaking an IoT deployment [9].

The target in this paper is to obtain an arithmetic framework aimed at generic edge computing environments, based on simple arithmetic operations in order to simplify routing and switching operations as much as possible, thus finding out the proper destination without having to look into either the routing tables for internetwork traffic or the switching tables for intranetwork flows, even though hardware and software implementations may influence which approach is more efficient. In that sense, the division algorithm provides an interesting capability when focusing on integer numbers because it may provide a quotient and a remainder, where the former may be used to identify an item being traversed and the latter may be employed to identify the entry or exit point out of such an item. Furthermore, a layered approach may make use of some variations of such parameters to adjust them accordingly to the particular setup of each layer.

Additionally, it is to be noted that such a layered model includes end devices at the bottom tier, also known as hosts, along with remote computing devices such as edge nodes, fog nodes and cloud nodes at the upper tiers, those following certain arithmetic rules related to the hosts located below them. Moreover, all those items involved are being interconnected through a wired environment whose port numbers follow certain arithmetic rules according to the hosts situated down below. In addition, wireless IoT devices might also take part in this framework by connecting to end devices, although the communications among such hosts will be undertaken through the wired paths defined by the arithmetic rules governing this framework.

**Figure 1:**
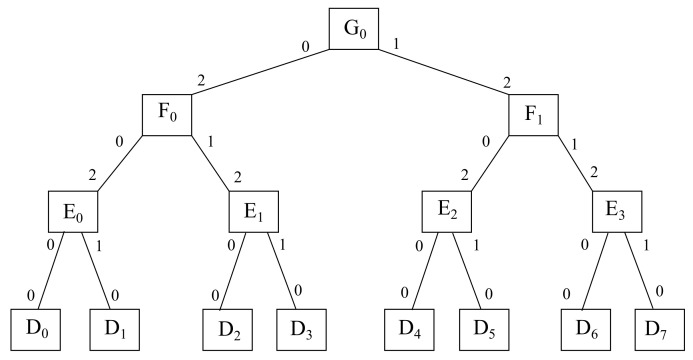
Instance of the arithmetic framework proposed.

In this sense, it might be reminded that the main point of this article is to define a simple arithmetic scheme based on integer divisions and modular arithmetic so as to identify each of the devices being traversed in the path between any given pair of end devices, as well as all the ports being involved in such a path. Therefore, it is important to define a determined numbering scheme in each layer, which is the sequential enumeration of items from left to right, along with a definite port scheme, which also goes left to right, starting with the downlink ports and carrying on with the uplink ports. Figure 1 exhibits an instance with the infrastructure proposed, even though the arithmetic expressions to move through it may be given later on. In addition, such expressions might be seen as an alternative way to current forwarding schemes based on searching for matches into the appropriate forwarding tables, those performing either routing or switching, depending whether the forwarding device works at layer 3 or at layer 2 according to the OSI model, which standardizes network communications.

Such a layered approach facilitates to break up the model into three different kinds of communications between any given pair of hosts, depending on how many hops away are the nearest common intermediate item to both of them. Additionally, three diverse approaches have been carried out in order to offer different options to deal with the model. It is to be said that those models have been verified by means of theorems and their proves related to arithmetic means, as well as by algebraic means with the help of an abstract process algebra named Algebra of Communicating Processes (ACP), which is branded as a formal description technique (FDT) [10].

The organization of the rest of the paper goes as follows: Section 2 presents the mathematical background needed to build up the models, afterwards, Section 3 exhibits the foundation of the models proposed, next, Section 4 exposes the features of the models proposed, then, Section 5 develops a logical approach so as to suggest some theorems and their proves related to the communications taking place in the models proposed, in turn, Section 6 depicts the specification and verification of the models by algebraic means, and eventually, Section 7 draws some final conclusions.

## 2. Mathematical Fundamentals for the MEC Framework Proposed

Prior to studying the logical structure of the model proposed, a classification of integer division kinds is presented, followed by an introduction to the division theorem. Afterwards, modular arithmetic basics are cited, and in turn, the most important theorem proof tools are listed, and with that in mind, there are enough tools to study the basic facts of the aforesaid model.

### 2.1. Types of Integer Divisions

Focusing on integer numbers, whose whole set is represented by Z, the division of a dividend (D) by a divisor (d) returns two numbers, such as a quotient (q) and a remainder (r), where the former accounts for the maximum number of whole units of *D* distributed into *d*, whilst the latter portrays the mismatch of *D* in relation to *d*, such as a surplus in case it is positive, or the shortage in case it is negative, or evenly distributed if it is zero.

It is to be said that the standard Euclidean division is the most common convention when dealing with division within the integer domain, where the remainder is always positive [11]. However, if the remainder is not zero, other options are available. In fact, programming languages usually employ diverse types of division so as to fit different situations, such as floored division, ceiled division, rounded division and truncated division.

On the one hand, regarding floor and ceiled actions, the former always obtains an integer less or equal than the corresponding floating point division, whilst the latter always obtains an integer greater or equal than floating point division. On the other hand, with respect to round and truncate actions, the former performs floor or ceiled actions depending on which one obtains the remainder with the least absolute value, whereas the latter portrays floor or ceiled actions so as to obtain a remainder with the same sign of the dividend. Furthermore, standard Euclidean division might be seen as applied floored division in case divisor is positive or ceiled division in case the divisor is negative.

Additionally, the other three hardly used conventions might be applied as they are opposite to some of those exposed, such as always attaining a negative remainder or the remainder having the greatest absolute value or, otherwise, the remainder having a diverse sign out of the dividend. Anyway, Table 1 depicts all those different options related to integer division, where each convention has its opposite one.

### 2.2. Division Theorem

Sticking to the set of integer numbers Z, the division theorem states that if dividend *D* is any integer number and divisor *d* is a positive number, then there exist unique integers, called the quotient *q* and the remainder *r*, such that D=d·q+r, where 0≤|r|<d, which will account for 0≤r<d when dealing with standard Euclidean divisions when d>0. In order to prove this theorem, uniqueness may be previously proved, which, in turn, may lead to prove its existence [12].

First of all, uniqueness may be proved by assuming a couple, $q_{1}$ and $r_{1}$, and another couple, $q_{2}$ and $r_{2}$, all of them being part of Z, where both couples are satisfying the conclusion, such as the former given by Equation (1) and the latter by Equation (2):

(1) $$D=d\cdot q_{1}+r_{1}\;,\;0\;\leq \;|r_{1}\left|\;<\;|d\right|$$

(2) $$D=d\cdot q_{2}+r_{2}\;,\;0\;\leq \;|r_{2}\left|\;<\;|d\right|$$

By comparing both equations, it results in Equation (3), which implies that the difference $r_{2}-r_{1}$ is just a multiple of *d*:

(3) $$d\cdot q_{1}+r_{1}=d\cdot q_{2}+r_{2}\;\Rightarrow \;d\cdot \left(q_{1}-q_{2}\right)=r_{2}-r_{1}$$

Taking into account that $r_{1}$ and $r_{2}$ range from 0 all the way to d−1, then the difference $r_{2}-r_{1}$ is lower in absolute value than *d*, which may be incorporated in the previous expression as shown in Equation (4). In this sense, it is to be reminded that if a remainder were equal or greater than |d|, then its related quotient ought to be higher in absolute value:

(4) $$|d\cdot \left(q_{1}-q_{2}\right)\left|=\right|r_{2}-r_{1}\left|<|d\right|$$

In the aforementioned expression, the only integer being multiple of *d* being smaller in absolute value than |d| is 0, hence $|d\cdot (q_{1}-q_{2})|=0$. However, *d* must be a positive number, thus it could not be zero, leading to $q_{1}-q_{2}=0$, which account for $q_{1}=q_{2}$. If this result is translated into Equation (3), then it is obtained that $r_{2}-r_{1}=d\cdot 0=0$, thus leading to the fact that $r_{1}=r_{2}$, which clearly shows the uniqueness expected.

Afterwards, existence may be proved by taking into consideration all integer multiples of *d*, such that {k·d:k∈Z}. As d≠0, those multiples are equally spaced along the real line. In this context, let us take an integer *a* located inside the interval given by two consecutive multiples of *d*, such as k·d≤a<(k+1)·d, such as k∈Z.

Additionally, if the term k·d is subtracted out of all terms, it results in 0≤a−k·d<d. At this point, by applying the definition of the remainder, which may be easily deducted from the division theorem as r=D−d·q, it results in Equation (5):

(5) 0≤r<b=|b|

This way, uniqueness has been proved in a first stage, and in turn, existence has been done at a second stage, which leads to the conclusion that the division theorem has been duly proved.

### 2.3. Notions of Modular Arithmetic

Taking into consideration that the values established in this model are all natural numbers N, which happens to be a subset of integer numbers Z where all its positive numbers along with zero are included, then the aforementioned results will also apply to the framework presented for facilitating the forwarding operations between end devices, edge servers and fog servers, which may also include the forward paths to cloud servers.

In that sense, it is to be seen that modular arithmetic works with modulo *d* residues, those being defined as the natural numbers from 0 all the way to d−1, which may be related to the remainders of the integer division by *d* [13]. In this sense, it may be said that the modulo *d* residue of *D* is r≡D(modd), which may also be calculated as the remainder of the integer division, such as r=D−d·q.

Likewise, modular arithmetic induces an equivalence relation called congruence when any set of given values share the same residue modulo *d*, which is usually represented by its canonical item, that being located within the range from 0 to d−1. In this context, two given values, *x* and *y*, are meant to be congruent modulo *d* if, and only if, (x−y)/d is an integer, whereas they are not congruent modulo *d* if the aforesaid result is not an integer.

### 2.4. Nomenclature for Integer Divisions and Modular Arithmetic

As the identifiers within the MEC framework proposed are within the domain of natural numbers N, integer divisions and floored divisions match, whereas ceiled divisions account for an additional unit added up to the former if its remainder is not zero, or it just matches the former otherwise. Anyway, integer divisions may be expressed as in expression (6), whilst ceiled divisions are performed as in Equation (7), whereas modular arithmetic operations may be performed as in expression (8):

(6) $$\mathrm{int}\left(D\;/\;d\right)\;=\;\left\lfloor D\;/\;d\right\rfloor \;=\;\frac{D-D_{|d}}{d}$$

(7) $$\left\lceil D\;/\;d\right\rceil \;=\;-\left\lfloor -D\;/\;d\right\rfloor \;=\;-\frac{D_{|d}-D}{d}$$

(8) $$D\;\mathrm{mod}\;d\;=\;D_{|d}\;=\;{\left(D_{|d}+d\right)}_{|d}$$

### 2.5. Theorem Proving

It may be said that a theorem proof may be seen as a sequence of statements, which are either assumed or otherwise follow from a previous statement by a rule of inference. It may be said that there are four basic styles of proof, even though some variations may also be applied:

- Direct proof, such as if we assume *P* is true, therefore *Q* must also be true. This method is stated as P⇒Q.
- Proof by contraposition, such as if we assume *Q* is false, therefore *P* must also be false. This method is denoted as ¬Q⇒¬P.
- Proof by contradiction, where a contradiction is searched for in order to deny a statement, or otherwise, that statement must be true.
- Proof by induction, where a basis step is proved, followed by an inductive step, which will imply that the statement must be true.

These are the main techniques to prove theorems by providing mathematical reasoning about the correctness of the sentences involved [14].

## 3. Basics of the MEC Framework Proposed

In order to build up a model for MEC communications, it is necessary to begin with the definition of the diverse layers, as well as denoting the different kind of communications taking part in the model depending on the number of layers being involved, along with the modeling of each item belonging to such layers.

### 3.1. Roles of Each Layer within the MEC Framework Proposed

**Figure 2:**
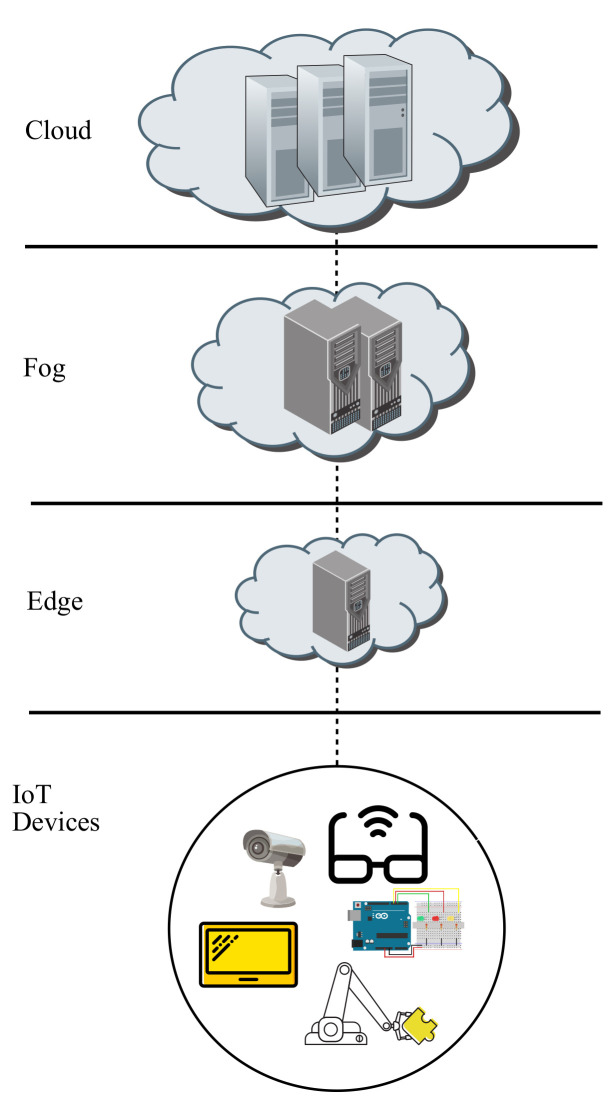
Layer hierarchy within the framework proposed.

The ever growing increment in MEC deployments may lead to try to search for a common infrastructure with a canonical number of items so as to facilitate the interconnections between the end devices tier and the different servers located at upper layers, namely, edge tier, fog tier and cloud tier, as exhibited in Figure 2.

The number of items within each layer may be normalized in a similar way as proposed in a fat tree architecture, which is a data centre architecture set up through three layers of switches. In this context, there is a parameter *k* influencing the whole layout, such as the number of items within each layer, along with the amount of interconnections between any two neighbouring layers, or the quantity of end hosts per end switch, per pod and overall [15].

Actually, the key point of the design proposed herein is to describe an infrastructure where the number of elements belonging to each layer depends on the value selected for parameter *k*, where each item in a given layer has a hub and spoke relationship with its *k* directly connected items in the neighboring lower layer. In this sense, the design will contain just $k^{0}=1$ cloud node (in case such a node is cited in the scenario proposed), $k^{1}=k$ fog nodes, $k^{2}$ edge nodes and $k^{3}$ end devices, where each individual item within any layer will be sequentially identified from left to right with a natural number going from 0 all the way to the predecessor of the correspondent limit value.

As shown in Figure 2, the layers involved in the MEC deployment layout proposed are Devices, Edge, Fog and Cloud. First of all, the role of the Devices layer is to either retrieve information from the environment through some attached sensors and pass them up to a particular server located on an upper layer or otherwise to act on the environment through some associated actuators according to the information provided by a given server situated on an upper layer.

Moreover, the role of the Edge layer is to furnish some remote computing resources powered by a certain AI, which will receive raw data sent over by a given source end device and will try to process them on its directly connected edge node, which will happen if the proper destination end device is also directly connected. If that is the case, then the processed data are forwarded back to that particular destination end device, whereas on the contrary, the raw data are forwarded up to the fog layer.

In addition, the role of the Fog layer is to supply some more powerful remote computing resources than the edge layer, as well as accounting for a more powerful AI, which will receive raw data being sent up from a given source edge server, that being directly connected to the source end device, and will try to process them on its directly connected fog node, which will occur if the appropriate destination edge node, that being linked to the destination end device, is also directly connected. If this is the case, then the processed data are sent back to that given destination edge server, whilst on the other hand, the raw data are sent up to the cloud layer.

Furthermore, the role of the Cloud layer is to grant even more powerful remote computing resources than the fog layer, including an even more powerful AI, which obtains the raw data being forwarded on from a source fog node and delivers the processed data being sent over to a particular destination fog node.

Depending on the layer where the server processing the data is located on, communications within the MEC framework proposed may be divided into three categories, such as intraedge if an edge node does it, intrafog if a fog node does it or interfog if a cloud node does it.

In summary, it is to be noted that the flow chart starts at a source end device reading some information from the environment through one of its sensors, which in turn, is passed on to a source edge server in a similar fashion as DNS queries are carried out, as server nodes become more powerful depending on the hierarchical layer they are being located, such that edge servers are less powerful than fog servers, whilst those are less powerful than cloud servers. Anyway, when a server succeeds in processing such raw data, those processed data are headed to the destination end device in order for one of its actuators to act on the environment.

With regards to the graphical representation of the items staying at each layer of the MEC framework proposed, it is to be reminded that device and edge layers are common to all scenarios presented, whereas fog and cloud layers differs on the approach stated. Therefore, the former are presented for all scenarios, whereas the latter are specified for each particular one.

### 3.2. Differences among the Three Scenarios Presented within the MEC Framework Proposed

As stated before, three diverse scenarios are going to be presented in relation to how interfog communications are carried out, where each of them maintain the same grounds for intraedge and intrafog communications.

The first one may be called ’spoke fog’, whose network topology for interfog traffic flows is hub and spoke, where a cloud node plays the role of hub and the all fog nodes play the role of spokes. In this case, two links are needed to go from a source fog server to a destination fog server, as the cloud node play the part of a meeting point to move among fog nodes.

The second one may be referred to as ’full mesh fog’, whose network topology for interfog communications is full mesh, which means that no cloud node is necessary. In that case, just one link is obviously needed to get from a source fog node to a destination fog node because there is always a path among any given pair of fog servers.

The third one may be named ’hybrid fog’, whose network topology for interfog paths includes both solutions exposed above, such as there is a cloud node in order to play a hub and spoke architecture, as well as n·(n−1)/2 links among fog nodes so as to cover all possible interfog paths as stated by full mesh network topologies. Therefore, this scenario may provide redundancy between both aforementioned solutions for interfog communications, thus presenting a more realistic scenario where different path strategies are considered in order to avoid single points of failure.

### 3.3. Representation of Items Located on Each Layer within the MEC Framework Proposed

**Figure 3:**
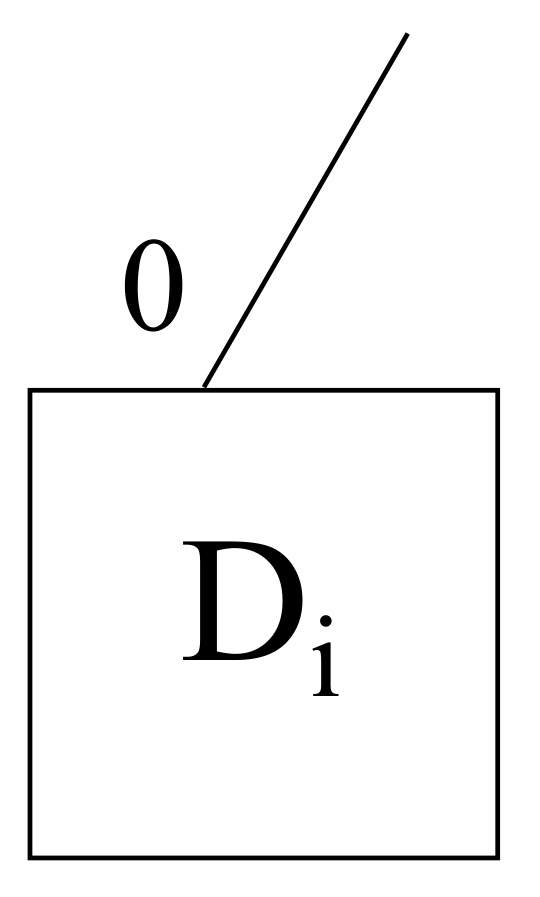
A generic device layout within the framework proposed.

Regarding devices for all scenarios, a given device $D_{i}$ with its unique own port 0 is shown in Figure 3.

**Figure 4:**
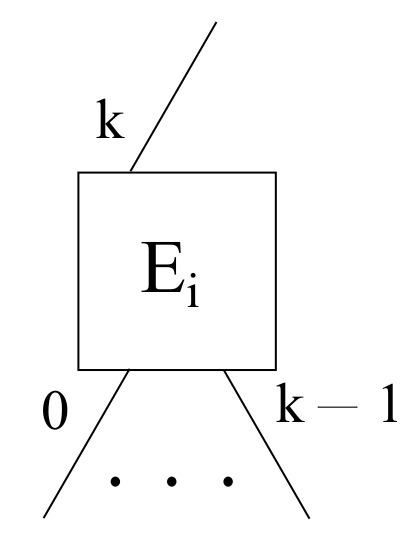
A generic edge node layout within the framework proposed.

With respect to edge servers for all scenarios, a particular edge node $E_{i}$ with its *k* downlink ports ranging from 0 to k−1, as well as its only uplink port *k* is shown in Figure 4, taking into account that downlink ports are first numbered from left to right, and afterwards, uplink ports carry on with the same numbering sequence also from left to right.

**Figure 5:**
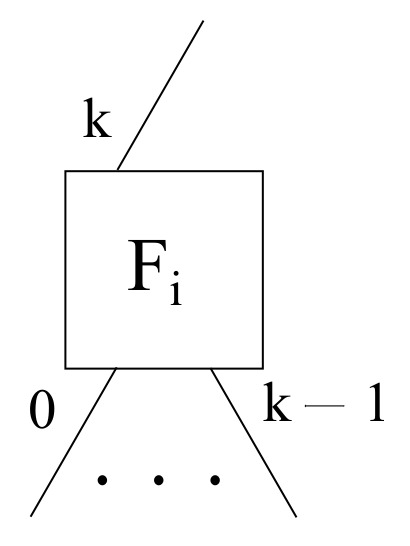
A generic fog node layout for the hub and spoke mode within the framework proposed.

Focusing on fog servers, three different scenarios have been presented, thus, three diverse layouts are needed. First of all, a spoke fog scenario requires that each given fog node $F_{i}$ has an analogous port setup as edge nodes, such that it needs its own *k* downlink ports, being labeled from 0 to k−1, as well as its sole uplink port, being labeled as *k*, in order to achieve a hub and spoke topology with the cloud layer, as exhibited in Figure 5.

**Figure 6:**
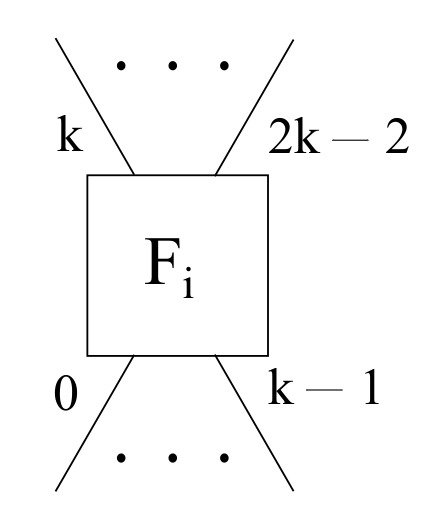
A generic fog node layout for the full mesh mode within the framework proposed.

Furthermore, a full mesh fog scenario has a link to the rest of k−1 fog nodes within the layout, whilst not having either any cloud node nor any uplink to those. Therefore, each given fog node *i* needs some *k* downlink ports going from 0 to k−1, along with some k−1 uplink ports ranging from *k* to 2k−2 in order to achieve a full mesh topology among all fog servers, as depicted in Figure 6.

**Figure 7:**
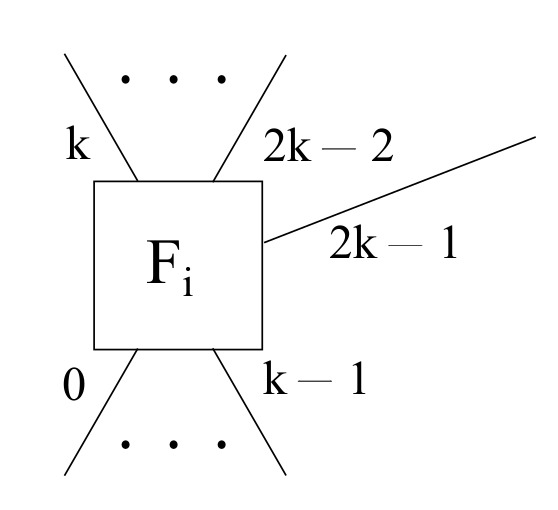
A generic fog node layout for the hybrid mode within the framework proposed.

Additionally, a hybrid fog scenario may be obtained by mixing together the hub and spoke and the full mesh scenarios in a single one. Hence, each particular fog node *i* has some *k* downlink ports ranging from 0 all the way to k−1, some k−1 uplink ports going from *k* to 2k−2 so as to attain full mesh topology among all fog nodes, and an extra uplink port 2k−1 in order to achieve a hub and spoke topology with the cloud layer, as exposed in Figure 7.

**Figure 8:**
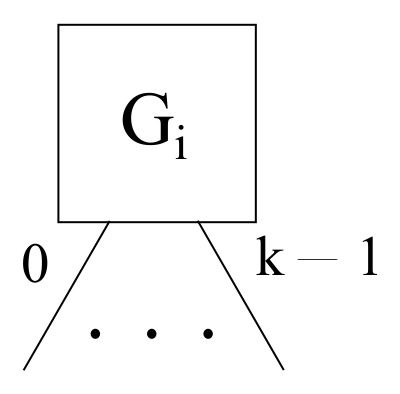
A generic cloud node layout within the framework proposed.

Eventually, a cloud node $G_{i}$ is necessary in the hub and spoke and hybrid scenarios, where a cloud node has *k* downlink ports ranging from 0 to k−1 aimed at the corresponding fog nodes, as shown in Figure 8.

## 4. Features of the MEC Framework Proposed

Taking all the above into consideration, some generic frameworks for MEC implementations are going to be proposed, which share the same approach for the interconnection of the three lower layer, but have different approaches for the interconnection of upper layers. This accounts for all approaches having the same intraedge and intrafog communication schemes, whilst having its own approaches for interfog communication schemes.

In this sense, it is to be noted that, in all case scenarios, variable *a* identifies the source host, whereas variable *b* identifies the destination host, whilst parameter *k* has been previously defined in the last section and states how many spokes are hanging on each item acting as a hub, located on the edge, fog and cloud layers. Moreover, those arithmetic expressions related to intermediate nodes and ports between the source host and the hub just employ *a* and *k*, whilst those referred between the hub and the destination host only utilize *b* and *k*, whereas those bypassing the role of a hub, specifically full mesh topology links among fog nodes, make use of the three variables.

### 4.1. Intraedge Scenario

**Figure 9:**
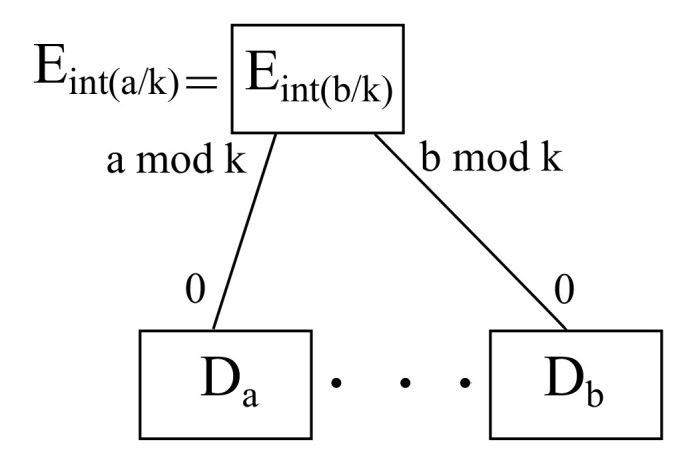
Schematic diagram for intraedge communication.

Sticking to intraedge communications, there is a common server at the edge layer, meaning that the source edge, being represented by $E_{\left\lfloor a/k\right\rfloor }$, is the same as the destination edge, being indicated by $E_{\left\lfloor b/k\right\rfloor }$, as it is denoted in Figure 9. On the other hand, the corresponding port to obtain the the relevant devices hanging on a shared edge (as the same edge connect to both source and destination devices) are given by $a_{|k}$ for its dowlink port pointing at the source device *a*, as well as $b_{|k}$ for its downlink port looking at the destination device *b*. Additionally, all devices have a unique port labeled as 0.

For instance, considering parameter k=2, if source host is a=0 and destination host is b=1, then the source edge is $\left\lfloor a\;/\;k\right\rfloor =\left\lfloor 0\;/\;2\right\rfloor =0$ and the destination edge is $\left\lfloor b\;/\;k\right\rfloor =\left\lfloor 1\;/\;2\right\rfloor =0$, meaning $\left\lfloor a\;/\;k\right\rfloor =\left\lfloor b\;/\;k\right\rfloor$, thus *a* and *b* are connected to the same edge node, hence intraedge communication takes place between *a* and *b*. Furthermore, the edge port $a_{|k}=0_{|2}=0$ connects to the source host a=0, whereas the edge port $b_{|k}=1_{|2}=1$ does so to the destination host b=1.

### 4.2. Intrafog Scenario

**Figure 10:**
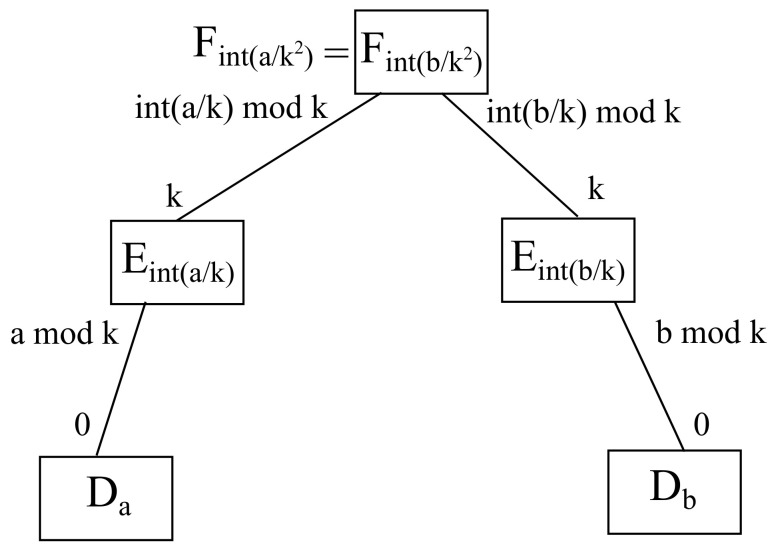
Schematic diagram for intrafog communication.

Moving to intrafog communications, there is a common server at the fog layer, resulting in the source fog, being indicated by $F_{\left\lfloor a/k^{2}\right\rfloor }$, being the same as the destination fog, being denoted by $F_{\left\lfloor b/k^{2}\right\rfloor }$, as it is shown in Figure 10. On the other hand, the proper port to reach the relevant devices linking on a share fog (because the same fog connect to both source and destination edges, which in turn, hang the source and destination devices, respectively) are stated by ${\left\lfloor a/k\right\rfloor }_{|k}$ for its downlink port looking at the source edge $E_{\left\lfloor a/k\right\rfloor }$, as well as ${\left\lfloor b/k\right\rfloor }_{|k}$ for its downlink port pointing at the destination edge $E_{\left\lfloor b/k\right\rfloor }$. Needless to say that the links between source edge and the source device, as well as the destination edge and the destination device, remain the same as exposed above.

For instance, considering parameter k=2, if source host is a=0 and destination host is b=2, then the source edge $\left\lfloor a\;/\;k\right\rfloor =\left\lfloor 0\;/\;2\right\rfloor =0$ differs from the destination edge $\left\lfloor b\;/\;k\right\rfloor =\left\lfloor 2\;/\;2\right\rfloor =1$, even though the source fog $\left\lfloor a\;/\;k^{2}\right\rfloor =\left\lfloor 0\;/\;2^{2}\right\rfloor =0$ matches the destination fog $\left\lfloor b\;/\;k^{2}\right\rfloor =\left\lfloor 2\;/\;2^{2}\right\rfloor =0$, meaning $\left\lfloor a\;/\;k^{2}\right\rfloor =\left\lfloor b\;/\;k^{2}\right\rfloor$, thus *a* and *b* are connected to the same fog node, hence intrafog communication takes place between *a* and *b*. Furthermore, the fog port ${\left\lfloor a\;/\;k\right\rfloor }_{|k}={\left\lfloor 0\;/\;2\right\rfloor }_{|2}=0$ connects to the source edge, namely, $\left\lfloor a\;/\;k\right\rfloor =\left\lfloor 0\;/\;2\right\rfloor =0$, which in turn is linked to host a=0, whereas the fog port ${\left\lfloor b\;/\;k\right\rfloor }_{|k}={\left\lfloor 2\;/\;2\right\rfloor }_{|2}=1$ does so to the destination edge, namely, $\left\lfloor b\;/\;k\right\rfloor =\left\lfloor 2\;/\;2\right\rfloor =1$, which in turn is connected to host b=2.

### 4.3. Interfog Scenario

Regarding interfog communications, two different strategies are going to be proposed herein, such as a hub and spoke interfog approach and a full mesh interfog approach. In both cases, there will be a source fog, being labeled as $F_{\left\lfloor a/k^{2}\right\rfloor }$, which is different of a destination fog, being denoted as $F_{\left\lfloor b/k^{2}\right\rfloor }$, but obviously, the path to go between them differs, as the former does it through a cloud server and the latter does it through a direct link. Additionally, a third strategy will be exposed as a combination of the aforesaid methods.

#### 4.3.1. Fog Spoke Approach

**Figure 11:**
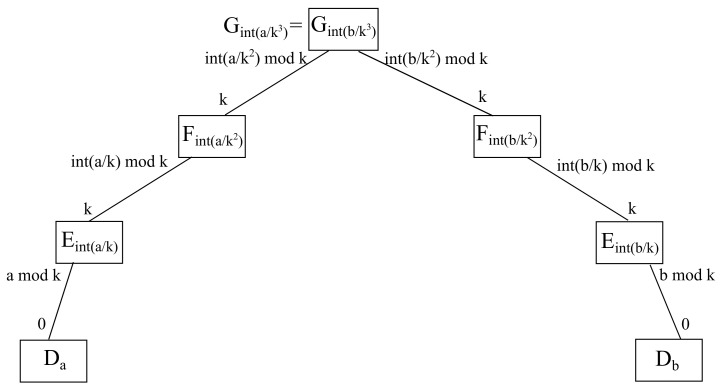
Schematic diagram for hub and spoke interfog communication.

It is to be considered that a cloud server is the meeting point through which all fog servers communicate to each other. With respect to such a cloud node, it is going to be unique in the whole infrastructure, hence, it may be calculated either as a source cloud, being denoted by $G_{\left\lfloor a/k^{3}\right\rfloor }$, or as a destination cloud $G_{\left\lfloor b/k^{3}\right\rfloor }$, as both expressions obviously match, as it is exhibited in Figure 11.

For instance, considering parameter k=2, if source host is a=0 and destination host is b=4, then the source edge $\left\lfloor a\;/\;k\right\rfloor =\left\lfloor 0\;/\;2\right\rfloor =0$ differs from the destination edge $\left\lfloor b\;/\;k\right\rfloor =\left\lfloor 1\;/\;2\right\rfloor =2$, as well as the source fog $\left\lfloor a\;/\;k^{2}\right\rfloor =\left\lfloor 0\;/\;2^{2}\right\rfloor =0$ differs from the destination fog $\left\lfloor b\;/\;k^{2}\right\rfloor =\left\lfloor 4\;/\;2^{2}\right\rfloor =1$. Thus, *a* and *b* are connected to different fog nodes, and hence interfog communication takes place between *a* and *b*. Alternatively, it happens that the source cloud $\left\lfloor a\;/\;k^{3}\right\rfloor =\left\lfloor 0\;/\;2^{3}\right\rfloor =0$ and the destination cloud $\left\lfloor b\;/\;k\right\rfloor =\left\lfloor 4\;/\;2^{3}\right\rfloor =0$, meaning $\left\lfloor a\;/\;k^{3}\right\rfloor =\left\lfloor b\;/\;k^{3}\right\rfloor$, which may also be seen as just one single cloud, thus accounting for intracloud communication. Furthermore, the cloud port ${\left\lfloor a\;/\;k^{2}\right\rfloor }_{|k}={\left\lfloor 0\;/\;2^{2}\right\rfloor }_{|2}=0$ connects to the source fog, namely, $\left\lfloor a\;/\;k^{2}\right\rfloor =\left\lfloor 0\;/\;2^{2}\right\rfloor =0$, which is linked to the source edge, namely, $\left\lfloor a\;/\;k\right\rfloor =\left\lfloor 0\;/\;2\right\rfloor =0$, which is further tied to source host a=0. Meanwhile, the cloud port ${\left\lfloor b\;/\;k^{2}\right\rfloor }_{|k}={\left\lfloor 4\;/\;2^{2}\right\rfloor }_{|2}=1$ does so to the destination fog, namely, $\left\lfloor b\;/\;k^{2}\right\rfloor =\left\lfloor 4\;/\;2^{2}\right\rfloor =1$, which is tied to the destination edge, namely $\left\lfloor b\;/\;k\right\rfloor =\left\lfloor 1\;/\;2\right\rfloor =1$, which is further linked to destination host b=4.

In addition, its downlink port going to the source fog $\left\lfloor a/k^{2}\right\rfloor$ is given by ${\left\lfloor a/k^{2}\right\rfloor }_{|k}$, whilst its downlink port looking at the destination fog $\left\lfloor b/k^{2}\right\rfloor$ is given by ${\left\lfloor b/k^{2}\right\rfloor }_{|k}$. On the other hand, the lower layer items and ports remain the same as stated above.

#### 4.3.2. Fog Full Mesh Approach

**Figure 12:**
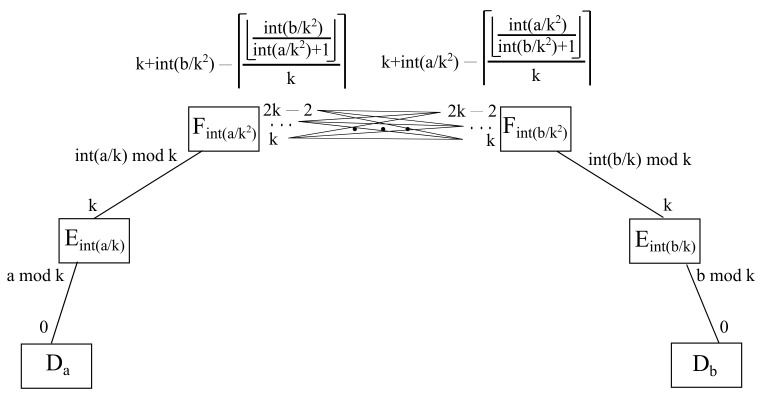
Schematic diagram for full mesh interfog communication.

In this case, there is no cloud server, as it is exhibited in Figure 12. Moreover, a source fog node and a destination fog node are obviously different items, even though they are directly connected because of the full mesh architecture. It is to be noted that each fog node needs k−1 uplink ports in order to be interconnected with all their k−1 fog node counterparts in order to achieve a full mesh topology [16].

On the other hand, any type of connection among fog nodes is feasible, such as k-ary n-cube or any other type of partial mesh scheme, even though full mesh has been selected herein for simplicity purposes. It is to be reminded that partial mesh may not have a direct link between any pair of fog nodes, which may make the design harder to be modeled, even though it might be faced in a future study.

Sticking to the full mesh scheme, the uplink port identifiers are always incremental with respect to the node on the other side of the channel, such that a link to the fog server with the lower identifier will be assigned port *k*, all the way to a link towards the fog node with the higher identifier will be associated to port 2k−2. On the other hand, the lower layer items and ports remain the same as exposed above.

For instance, considering parameter k=2, if source host is a=2 and destination host is b=5, then the source edge $\left\lfloor a\;/\;k\right\rfloor =\left\lfloor 2\;/\;2\right\rfloor =1$ differs from the destination edge $\left\lfloor b\;/\;k\right\rfloor =\left\lfloor 5\;/\;2\right\rfloor =2$, as well as the source fog $\left\lfloor a\;/\;k^{2}\right\rfloor =\left\lfloor 2\;/\;2^{2}\right\rfloor =0$ differs from the destination fog $\left\lfloor b\;/\;k^{2}\right\rfloor =\left\lfloor 5\;/\;2^{2}\right\rfloor =1$. Thus, *a* and *b* are connected to different fog nodes, and hence interfog communication takes place between *a* and *b*. As there is no cloud node in this case scenario, then the link between source fog node $\left\lfloor a\;/\;k^{2}\right\rfloor =\left\lfloor 2\;/\;2^{2}\right\rfloor =0$ and destination fog node $\left\lfloor b\;/\;k^{2}\right\rfloor =\left\lfloor 5\;/\;2^{2}\right\rfloor =1$ is being used, where its source port in the former and its destination port in the latter are defined next.

**Figure 13:**
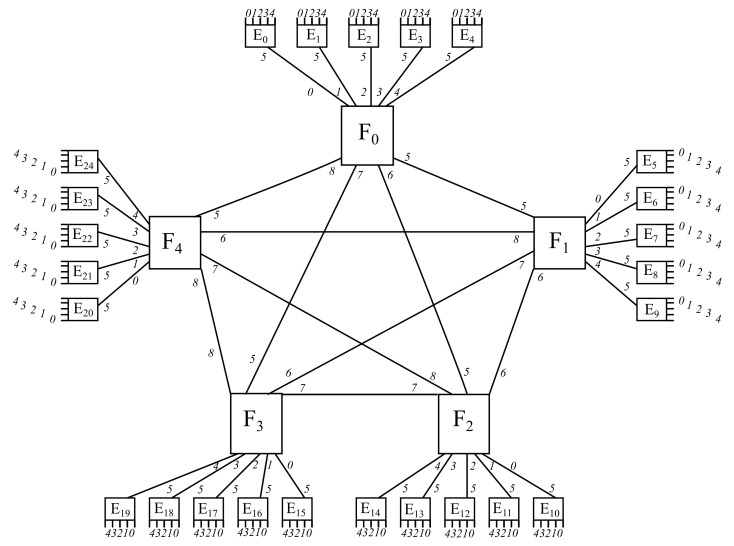
Port layout for full mesh fog communication when k=5.

In this sense, the uplink port layout to achieve the full mesh fog communications for each of the fog nodes involved are ordered in an incremental manner, such that the link to the lowest fog identifier is branded as *k*, the second lowest one is labeled as k+1, and so on, until the highest on is named as 2k−2, as it is shown in Figure 13. On the contrary, the downlink port layout connects each port from 0 to k−1 to the linked edge node whose remainder of its division by *k* obtains such a port, which may also be referred to a given host *h* hanging on them as the remainder of the division of $\left\lfloor h\;/\;k\right\rfloor$ by *k*.

It is also to be considered that a port going to itself is not permitted, as it would not make any sense in this context, so it must always be skipped out, which provokes that source ends of links towards a destination fog node being identified with a lower natural number than the source fog node result in $k+\left\lfloor b\;/\;k^{2}\right\rfloor$. In case it is a higher natural number, it needs to apply a correction factor of −1, such as in $k+\left\lfloor b\;/\;k^{2}\right\rfloor -1$. Luckily, both expressions may be collapsed in just one being useful in both cases by substituting −1 with a special corrector factor included in expression (9), which achieves the expected results in both cases:

(9) $$\mathrm{Source}\;\mathrm{port}\;\mathrm{in}\;\mathrm{full}\;\mathrm{mesh}=k+F_{\left\lfloor b\;/\;k^{2}\right\rfloor }-\left\lceil \frac{\left\lfloor F_{\left\lfloor b/k^{2}\right\rfloor }\;/\;(F_{\left\lfloor a/k^{2}\right\rfloor }+1)\right\rfloor }{k}\right\rceil$$

Analogously, the destination end of each interfog link carries similar features, where destination ends of links towards source nodes being identified with a higher natural number than the destination fog is given by $k+\left\lfloor a\;/\;k^{2}\right\rfloor$, whereas if it is a lower natural number, it requires to apply a corrector factor of −1, such as $k+\left\lfloor a\;/\;k^{2}\right\rfloor -1$. Fortunately, both expressions may be comprised in only one being ready to use in both cases by substituting −1 with another term with a special corrector factor included in Equation (10), which attains the expected outcome in both cases.

(10) $$\mathrm{Destination}\;\mathrm{port}\;\mathrm{in}\;\mathrm{full}\;\mathrm{mesh}=k+F_{\left\lfloor a\;/\;k^{2}\right\rfloor }-\left\lceil \frac{\left\lfloor F_{\left\lfloor a/k^{2}\right\rfloor }\;/\;(F_{\left\lfloor b/k^{2}\right\rfloor }+1)\right\rfloor }{k}\right\rceil$$

It is to be mentioned that both expressions take advantage of fog nodes being identified as members of the group $Z_{k}$, those being formed by the set of natural numbers going from 0 all the way to k−1. Hence, if source fog is identified by $i=F_{\left\lfloor a\;/\;k^{2}\right\rfloor }$ and destination fog is identified by $j=F_{\left\lfloor b\;/\;k^{2}\right\rfloor }$, it is to be said that if i>j, those representing both ends of an interfog channel, then the term with the floored division $\left\lfloor i\;/\;\left(j+1\right)\right\rfloor$ results in a positive natural number, or zero otherwise, whereas if i<j, then the term with the floored division $\left\lfloor j\;/\;\left(i+1\right)\right\rfloor$ results in a positive natural number, or zero otherwise.

Afterwards, all those positive natural numbers obtained above are normalized to 1 by means of applying the ceiled division by *k*, such that $\left\lceil \left\lfloor i\;/\;\left(j+1\right)\right\rfloor /k\right\rceil$ or $\left\lceil \left\lfloor j\;/\;\left(i+1\right)\right\rfloor /k\right\rceil$. It is to be noted that *k* is greater than any possible of the results above, as there are *k* fog nodes, thus being identified from 0 to k−1. Moreover, it is needless to say that zero is invariant with respect to normalization, so it sticks to zero. In addition, the addition of an extra unit to the denominators of all those fractions is just to avoid the potential division by zero and it does not affect the final outcome in any way.

Therefore, the ceiling division is applied to the whole fraction in order to achieve either 1 or 0, thus obtaining the corrector factor whenever is needed with just one single term in order to attain the corresponding port. It is to be remarked that *i* has been substituted by $\left\lfloor a\;/\;k^{2}\right\rfloor$, whilst *j* has been substituted by $\left\lfloor b\;/\;k^{2}\right\rfloor$ in the aforementioned expressions.

#### 4.3.3. Fog Hybrid Approach

**Figure 14:**
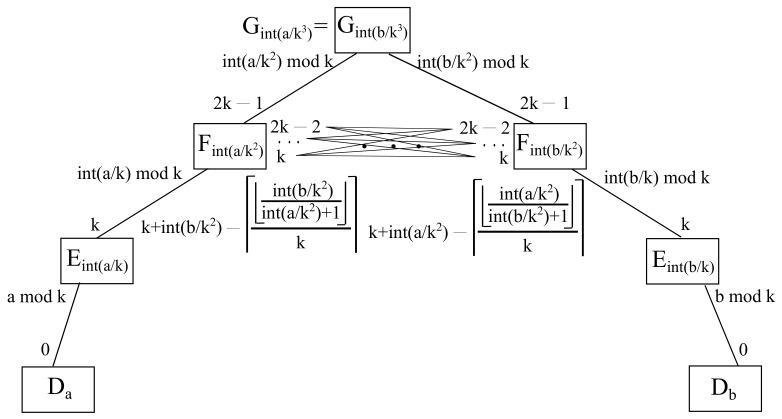
Schematic diagram for hybrid interfog communication.

Basically, this scenario is a mix of those previously proposed, hence it has a single cloud server at its highest layer, with *k* fog servers connected to the cloud in a hub and spoke manner, whilst all those fog nodes are also interconnected in a full mesh fashion. Furthermore, each fog server has *k* edge servers hanging on it, which accounts for an overall amount of $k^{2}$ of such servers. Additionally, each edge server has *k* IoT devices hanging on it, which represents $k^{2}$ of such devices below any fog node and a total amount of $k^{3}$ overall. This topology is shown in Figure 14, which provides some redundant paths just in case of a relevant failure in the system or alternative path for applying load balancing policies.

### 4.4. Relevant Number of Items and Links in Each Interfog Scenario

For clarification purposes, Table 2 compiles all relevant values related to *k* for each kind of layer in the intracloud scenario.

Otherwise, Table 3 summarizes the relevant values referred to *k* for each sort of layer in the full mesh scenario.

On the other hand, regarding links within each topology, it may be seen that each hub and spoke topology needs as many links as its amount of connected spokes, which accounts for *k* in the layout proposed. Therefore, as there are $1+k+k^{2}$ hub and spoke topologies in the intracloud scenario, where the 1 value is related to the cloud node, the *k* value is related to the fog nodes and the $k^{2}$ value is related to the edge nodes. Considering that each one has *k* links, then the overall amount of links is $k+k^{2}+k^{3}$.

However, in the full mesh scenario, there are $k+k^{2}$ hub and spoke topologies, where the *k* value is related to the fog nodes, whilst the $k^{2}$ value is related to the edge nodes. Additionally, there are k·(k−1)/2 interfog connections, thus the total number of links is $k+k^{2}+k^{2}\;/\;2+-k\;/\;2$, which accounts for $k\;/\;2+3k^{2}\;/\;2$.

On the contrary, in the hybrid scenario, there are $k+k^{2}+k^{3}+k\cdot (k-1)\;/\;2$, which equals $k\;/\;2+3k^{2}\;/\;2+k^{3}$.

## 5. Logical Approach to the MEC Framework Proposed

The aforesaid expressions cited in the figures above may be also deduced by logical means, in a way that some theorems may be quoted focused on the MEC framework proposed, followed by the corresponding proofs to those theorems.

### Theorems Applied to the Framework Proposed

Taking into account that the framework proposed works with natural numbers as identifiers for the different items located on the diverse layers within the model, it is possible to find every intermediate device, forming the shortest path between a source device and a destination device, as well as every single port being traversed in any device taking place in such a communication.

It is to be remembered that the infrastructure heavily depends on parameter *k*, which may induce the use of integer divisions by *k*, or otherwise, the modular arithmetic modulo *k*, as suggested in the figures presented above. The former may well be used so as to establish the item identification of the intermediate devices (servers located at the edge layer, at the fog layer or at the cloud layer), whereas the latter may well be employed to spot the port identification within each of the intermediate devices being traversed on the way from a source device *a* to a destination device *b*.

The expressions for each case scenario have already been exposed in the figures above, although those may also be deducted from the following theorems, which are all duly proved by applying the division theorem presented above.

**Theorem 1.**  *All intraedge communications take place within the ports of a given edge node in any given scenario.*

**Proof of Theorem 1.**  Considering a host *h*, it is connected to an edge node $\left\lfloor h\;/\;k\right\rfloor$, as there are *k* hosts per edge node. Hence, intraedge communications meet the condition $\left\lfloor a\;/\;k\right\rfloor =\left\lfloor b\;/\;k\right\rfloor$, meaning that a source host *a* and a destination host *b* share the same edge node. Consequently, due to the uniqueness and existence of each available remainder, that edge node may have a downlink port going towards *a*, namely, $a_{|k}$, as well as another one heading for *b*, namely, $b_{|k}$. Therefore, there is always a path between *a* and *b* when it comes to intraedge communications. □

**Theorem 2.**  *All intrafog communications take place within the ports of a given fog node in any given scenario.*

**Proof of Theorem 2.**  Considering a host *h*, it is connected to an edge node $\left\lfloor h\;/\;k\right\rfloor$, whilst such an edge node is connected to a fog node $\left\lfloor h\;/\;k^{2}\right\rfloor$, as there are always *k* hosts per intermediate node. Hence, intrafog communications fulfill the condition $\left\lfloor a\;/\;k^{2}\right\rfloor =\left\lfloor b\;/\;k^{2}\right\rfloor$, whilst $\left\lfloor a\;/\;k\right\rfloor \neq \left\lfloor b\;/\;k\right\rfloor$, meaning that a source host *a* and a destination host *b* share the same fog node, but not the same edge node. Consequently, due to the uniqueness and existence of each available remainder, that fog node may have a downlink port going towards the edge node where *a* is hanging on, namely, ${\left\lfloor a\;/\;k\right\rfloor }_{|k}$, as well as another one heading for the edge node where *b* is connected, namely, ${\left\lfloor b\;/\;k\right\rfloor }_{|k}$, where in both cases the uplink port on the edge side is *k*. On the other hand, communication between the source host *a* and its source edge server $\left\lfloor a\;/\;k\right\rfloor$ are carried out through its downlink port $a_{|k}$, whilst communication between the destination host *b* and its destination edge server $\left\lfloor b\;/\;k\right\rfloor$ are undertaken through its downlink port $b_{|k}$. Therefore, there is always a path between *a* and *b* when it comes to intrafog communications. □

**Theorem 3.**  *All interfog communications take place within the ports of the cloud node in the fog spoke scenario.*

**Proof of Theorem 3.**  Considering a host *h*, it is connected to an edge node $\left\lfloor h\;/\;k\right\rfloor$, whilst such an edge node is connected to a fog node $\left\lfloor h\;/\;k^{2}\right\rfloor$, whereas such a fog node is connected to a cloud node $\left\lfloor h\;/\;k^{3}\right\rfloor$, as there are always *k* hosts per intermediate node. Hence, interfog communications fulfill the condition $\left\lfloor a\;/\;k^{3}\right\rfloor =\left\lfloor b\;/\;k^{3}\right\rfloor$, whilst $\left\lfloor a\;/\;k^{2}\right\rfloor \neq \left\lfloor b\;/\;k^{2}\right\rfloor$, which implies $\left\lfloor a\;/\;k\right\rfloor \neq \left\lfloor b\;/\;k\right\rfloor$, meaning that a source host *a* and a destination host *b* share the same cloud node, but not the same fog node, which also involves different edge nodes. Consequently, due to the uniqueness and existence of each available remainder, that cloud node may have a downlink port going towards the fog node where *a* is hanging on, namely ${\left\lfloor a\;/\;k^{2}\right\rfloor }_{|k}$, as well as another one heading for the edge node where *b* is connected, namely, ${\left\lfloor b\;/\;k^{2}\right\rfloor }_{|k}$, where in both cases the uplink port on the fog side is *k* if the intracloud scenario is contemplated. On the other hand, communication between the source host *a* and its source edge server $\left\lfloor a\;/\;k\right\rfloor$ are carried out through its downlink port $a_{|k}$, whilst communication between such a source edge node and the source fog node $\left\lfloor a\;/\;k^{2}\right\rfloor$ are made through its downlinks port ${\left\lfloor a\;/\;k\right\rfloor }_{|k}$. Likewise, communications between the destination host *b* and its destination edge server $\left\lfloor b\;/\;k\right\rfloor$ are undertaken through its downlink port $b_{|k}$, whereas communication between such a destination edge node and the destination fog node $\left\lfloor b\;/\;k^{2}\right\rfloor$ are made through its downlinks port ${\left\lfloor b\;/\;k\right\rfloor }_{|k}$. Therefore, there is always a path between *a* and *b* when it comes to interfog communications when dealing with the fog spoke scenario, also known as intracloud communications. □

**Theorem 4.**  *All interfog communications take place within the uplink ports of the fog nodes involved in the fog full mesh scenario.*

**Proof of Theorem 4.**  The only difference with the previous case is the interfog communications, which work in a full mesh fashion according to the expressions given in the last section, where every single fog node has a straight link with all the rest of fog nodes, such as the source port of such a link in the source fog node given by $k+F_{\left\lfloor b\;/\;k^{2}\right\rfloor }-\left\lceil \frac{\left\lfloor F_{\left\lfloor b/k^{2}\right\rfloor }\;/\;(F_{\left\lfloor a/k^{2}\right\rfloor }+1)\right\rfloor }{k}\right\rceil$, whereas the destination port of such a link in the destination fog node is stated by $k+F_{\left\lfloor a\;/\;k^{2}\right\rfloor }-\left\lceil \frac{\left\lfloor F_{\left\lfloor a/k^{2}\right\rfloor }\;/\;(F_{\left\lfloor b/k^{2}\right\rfloor }+1)\right\rfloor }{k}\right\rceil$. Therefore, there is always a path between *a* and *b* when it comes to interfog communications when dealing with the fog full mesh scenario. □

**Theorem 5.**  *All interfog communications take place within the ports of the cloud node in the fog hybrid scenario.*

**Proof of Theorem 5.**  This case scenario considers both fog spoke and fog full mesh, where both of them always provide a path between *a* and *b*, therefore, there is always a path between those hosts. □

## 6. Algebraic Modeling with ACP

Once the proper expressions have been defined for all nodes and their corresponding ports, it is time to model the three case scenarios proposed so as to find out whether their external behavior is the expected one [17]. In order to do that, a timeless process algebra called Algebra of Communicating Processes (ACP) is going to be employed because it is an abstract algebra just focusing on how each entity within the model acts on a regular basis, which allows to abstract away from the real nature of such entities.

Hence, ACP may focus just on the relationships established among the entities being involved in the model, thus permitting to mask the internal behavior of the model, whilst allowing to extract its external behavior, which may be defined as how an external observer may perceive the behavior of a model. On the other hand, ACP does not take time into account, which permits focusing on qualitative features as opposed to quantitative ones being derived from a time scale [18].

This section about algebraic modeling with ACP tries to apply the aforementioned theorems and expressions to algebraic notation so as to first describe the behavior of the entities involved in an algebraic manner, which then leads to obtaining the sequence of events of the whole model, which in turn unveils the external behavior of the model. Such descriptions are carried out by quoting the intermediate devices and their ports involved by means of the arithmetic expression being exposed in the previous sections when tracing the optimal path between a source host *a* and a destination host *b*, as all of them are influenced by the values of *a*, *b* and *k*.

Regarding ACP syntax and semantics, it may be said that there are two atomic actions, such as send and read [19], which might be compared to generic functions. Those actions are carried out by any pair of items, also known as entities, having a common unidirectional channel between them, where the send action is performed by the source entity through its source end of such a channel, and the read action is performed by the destination entity through its destination end of the same channel.

With respect to the messages flowing from the source to the destination of a given channel, those are usually described as *d*, as an acronym of data, and they are not really relevant. However, it is important to uniquely identify each unidirectional channel so as to be able to check whether communication may arise therein, which takes place when the send action is executed at the source end and the read action is run at the destination end in a concurrent manner.

The most common way to identify a channel is with a single variable, in a way that a common identifier is used at both ends of a link. However, for the purpose of using relevant identifiers for both ends of a single channel, it seems more interesting to describe each of its end with the set item-port, that being specified as the pair Item{Port} in the sending end (where the former is the element located at one end of a given channel and the latter is the starting point of the unidirectional channel being described) be and the pair {Port}Item in the receiving end (where the former is the ending point of the unidirectional channel being described and the latter is the element located at the other end of such a channel).

That way, if communication takes place in such a channel, it may be denoted by quoting both sets of item-ports involved, meaning those being located at both ends of such a channel, resulting in Item{Port} → {Port}Item. Therefore, the expressions exposed in Section 4 and Section 5, which are summarized in Table 4, are needed to specify the different item-port sets being present throughout the topologies described herein, which involve working with natural numbers and applying arithmetic operations.

Therefore, sending a given message *d* through a certain channel will be denoted by $s_{item\{port\}}\left(d\right)$, whilst reading a particular message *d* out of a certain channel will be indicated by $r_{\{port\}item}\left(d\right)$. This way, the former implies that the message exits out of a given item through a certain port, whereas the latter does that the message gets through a certain port into a given item. Moreover, communication is described by $c_{item\{port\}\to \{port\}item}\left(d\right)$ and covers the pass of information from source to destination.

Furthermore, the atomic actions being performed by an entity may relate among them by means of a set of operators, such as the sequential one, which is stated by ·, the alternate one, which is indicated by +, the concurrent one, which is established by ∣∣, or the conditional one, which is described as (True◃condition▹False) [20].

Hence, the behavior of an entity may be described by means of an algebraic expression, showing the concatenation of atomic actions along with the appropriate operators, which usually exhibits recursivity in order to portray a never-ending cycle. In addition, a sequence of events may be achieved running all the expressions describing a model in a concurrent manner, which may further lead to obtaining of the external behavior of such a model.

Additionally, the encapsulation operator, which is denoted by $\partial_{H}$, will force all internal atomic actions into either communication if there is a send action at one end of a channel and a read action at the other one, or otherwise they go deadlock, thus obtaining a sequence of events due to the interaction of all entities being part of the system [21]. At a later stage, the abstraction operator, which is described by $\tau_{I}$ will mask both internal actions and internal communications, hence allowing only the external atomic actions, thus unveiling the external behavior of the model [22]. At that point, the external behavior of the real system may also be worked out, and if both external behaviors share the same string of actions and the same branching structure, it may be concluded that they are both rooted branching bisimilar, which is a sufficient condition to have a model verified [23].

Regarding the channels available within the model, up to seven channels may be defined, where just the fog hybrid model will use all of them, as the fog spoke one will employ the cloud channels but not the fog-to-fog channels, and the fog full mesh one will do so the other way around. Anyway, Table 4 summarizes such channels, where the nomenclature of each channel is given by detailing the source item followed by the source end in curly brackets, then a right arrow signaling the direction of such a channel and, in turn, the destination end in curly brackets and the destination end. Furthermore, Table 5 states which channels are used in each of the three models studied. In summary, it is worth saying that the relationships between the intermediate devices and their ports involved when moving from source host *a* and destination host *b* are quoted in Table 4, whereas the channels used in each of the models proposed are cited in Table 5.

On the other hand, artificial intelligence may be applied at all intermediate nodes, resulting in edge AI, fog AI and cloud AI, where they are obviously incrementally more powerful. Hence, decision making related to packet forwarding so as to guide traffic flows from a given source to their indented destination may be denoted at those levels in the form of ${\mathrm{AI}}_{\mathrm{edge}}$, ${\mathrm{AI}}_{\mathrm{fog}}$ and ${\mathrm{AI}}_{\mathrm{cloud}}$, respectively. This way, raw traffic data before processing are represented by (d), whilst after processing they are represented by (e).

In the context of this paper, it is to be said that the communication approach undertaken herein does not need AI in any way to move traffic among end hosts, as the different channels forming the path from a source host a to a destination host b may be easily found out by applying the arithmetic expressions cited above. However, in order to give a more generic view of the application of this arithmetic framework, AI has been included in all intermediate devices, such as AI edge, AI fog or AI cloud, which might imply that other type of processing could be performed in such nodes.

Therefore, at this point the models for the three case scenarios are going to be represented by means of ACP. It is to be noted that each of the three scenarios proposed are being model with ACP following three stages, where the first one involves the algebraic models of each type of entity being present in such a model (*D* for devices, *E* for edges, *F* for fogs and *G* for clouds), then the second one involves running all entities concurrently, which shows up the sequence of events happening in the model, and the third one involves obtaining the external behavior of the model, which in turn is compared with the external behavior of the real system in order to obtain the verification of the model according to ACP rules.

### 6.1. Fog Spoke Scenario

First of all, the four entities involved in this scenario are going to be modelled so as to describe their behavior in an algebraic fashion, such as devices (*D*), edge nodes (*E*), fog nodes (*F*) and the cloud node (*G*), whereas *p* denotes any downlink port within an edge server, *q* denotes fog servers and *u* denotes the cloud server.

In this sense, recursive Equation (11) states that a particular device $D_{x}$ may either receive (*r*) any message (*d*) through its port 0 or send (*s*) any message (*d*) through its port 0 and will keep doing that forever, which is expressed by means of recursivity (thus executing $D_{x}$ indefinitely). It is to be noted that such an equation describes the behavior of any device within the topology, those going from 0 to $k^{3}-1$, as there are up to $k^{3}$ devices overall within the topology proposed.

On the other hand, recursive Equation (12) denotes that a particular edge node $E_{y}$, those going from 0 to $k^{2}-1$, may either receive a message through any of its lower ports (*p*), which will be forwarded down towards the destination device if intraedge communication takes place, or otherwise, that message is sent up towards its upper port *k*. In addition, if a message is coming from its upper port, that messages is forwarded down towards the destination device.

Similarly, recursive Equation (13) states that a given fog node $F_{z}$, those going from 0 to k−1, may either receive a message through any of its lower ports (*q*), which will be sent down towards the destination edge node if intrafog communication arises, or otherwise, that message is forwarded up towards its upper port *k*. Moreover, if a message is coming down its upper port, such a message is sent down towards the destination edge node.

Furthermore, recursive Equation (14) indicates that if a message is received through any port (*u*) located in the only cloud node *G*, such a message is forwarded down through the destination fog node. As stated above, some AI processing has also been include in the previous expressions in order to include any kind of processing with the incoming messages *d*, which might be considered as raw data, in order to be converted into processed data, those being denoted by outgoing messages *e*:

(11) $$D_{x}=\sum_{x=0}^{k^{3}-1}\left(r_{\left\{0\right\}D_{x}}\left(d\right)+s_{D_{x}\left\{0\right\}}\left(d\right)\right)\cdot D_{x}$$

(12) $$\begin{array}{c} E_{y}=\sum_{y=0}^{k^{2}-1}\sum_{p=0}^{k-1}(r_{\left\{p\right\}E_{y}}\left(d\right)\cdot \left({\mathrm{AI}}_{\mathrm{edge}}\cdot s_{E_{y}\left\{b_{|k}\right\}}\left(e\right)◃\;\left\lfloor a\;/\;k\right\rfloor =\left\lfloor b\;/\;k\right\rfloor \;▹s_{E_{y}\left\{k\right\}}\left(d\right)\right)+\\ r_{\left\{k\right\}E_{y}}\left(e\right)\cdot s_{E_{y}\left\{b_{|k}\right\}}\left(e\right))\cdot E_{y} \end{array}$$

(13) $$\begin{array}{c} F_{z}=\sum_{z=0}^{k-1}\sum_{q=0}^{k-1}(r_{\left\{q\right\}F_{z}}\left(d\right)\cdot \left({\mathrm{AI}}_{\mathrm{fog}}\cdot s_{F_{z}\left\{{\left\lfloor b\;/\;k\right\rfloor }_{|k}\right\}}\left(e\right)◃\;\left\lfloor a\;/\;k^{2}\right\rfloor =\left\lfloor b\;/\;k^{2}\right\rfloor \;▹s_{F_{z}\left\{k\right\}}\left(d\right)\right)+\\ r_{\left\{k\right\}F_{z}}\left(e\right)\cdot s_{F_{z}\left\{{\left\lfloor b\;/\;k\right\rfloor }_{|k}\right\}}\left(e\right))\cdot F_{z} \end{array}$$

(14) $$G=\sum_{u=0}^{k-1}\left(r_{\{u\}G}\left(d\right)\cdot {\mathrm{AI}}_{\mathrm{cloud}}\cdot s_{G\{{\left\lfloor b\;/\;k^{2}\right\rfloor }_{|k}\}}\left(e\right)\right)\cdot G$$

At this stage, the encapsulation operator may be applied in order to obtain a sequence of events within the model of intermediate items, thus considering devices as external items, which is exhibited in Equation (15).

It is to be noted that the destination ports have been completely described in the aforesaid models, although their corresponding intermediate destination items have been cited generically (by means of variables *y* and *z*), whereas all intermediate source items and their appropriate source ports have also been quoted generically.

However, in this context, all the intermediate remote servers involved in a certain communication, no matter whether they are located either on the edge, fog or cloud layers, may be easily spotted by means of the appropriate expressions depending on *a*, *b* and *k*, as exposed in Table 4, whilst likewise, their source downlink ports involved, namely, *p*, *q* and *u*, may also be determined therein:

(15) $$\begin{array}{c} \sum_{y=0}^{k^{2}-1}\sum_{z=0}^{k-1}\partial_{H}\left(E_{y}||F_{z}||G\right)=(r_{\left\{a_{|k}\right\}E_{\left\lfloor a\;/\;k\right\rfloor }}\left(d\right)\cdot ({\mathrm{AI}}_{\mathrm{edge}}◃\;\left\lfloor a\;/\;k\right\rfloor =\left\lfloor b\;/\;k\right\rfloor \;▹\\ c_{E_{\left\lfloor a\;/\;k\right\rfloor }\left\{k\right\}\to \left\{{\left\lfloor a\;/\;k\right\rfloor }_{|k}\right\}F_{\left\lfloor a\;/\;k^{2}\right\rfloor }}\left(d\right)\cdot ({\mathrm{AI}}_{\mathrm{fog}}◃\;\left\lfloor a\;/\;k^{2}\right\rfloor =\left\lfloor b\;/\;k^{2}\right\rfloor \;▹\\ c_{F_{\left\lfloor a\;/\;k^{2}\right\rfloor }\left\{k\right\}\to \left\{{\left\lfloor a\;/\;k^{2}\right\rfloor }_{|k}\right\}G_{\left\lfloor a\;/\;k^{3}\right\rfloor }}\left(d\right)\cdot {\mathrm{AI}}_{\mathrm{cloud}}\cdot c_{G_{\left\lfloor b\;/\;k^{3}\right\rfloor }\left\{{\left\lfloor b\;/\;k^{2}\right\rfloor }_{|k}\right\}\to \left\{k\right\}F_{\left\lfloor b\;/\;k^{2}\right\rfloor }}\left(e\right))\cdot \\ c_{F_{\left\lfloor b\;/\;k^{2}\right\rfloor }\left\{{\left\lfloor b\;/\;k\right\rfloor }_{|k}\right\}\to \left\{k\right\}E_{\left\lfloor b\;/\;k\right\rfloor }}\left(e\right))\cdot s_{E_{\left\lfloor b\;/\;k\right\rfloor }\left\{b_{|k}\right\}}\left(e\right))\cdot \partial_{H}\left(E_{y}||F_{z}||G\right) \end{array}$$

At this point, the abstraction operator may be applied so as to attain the external behavior of the model. Hence, if only external actions prevail, those are either receiving a message in a node located at the edge layer whose downlink port is coming from source host *a*, or otherwise, sending a message from another node situated at such a layer whose downlink port is going towards destination host *b*, as shown in Equation (16):

(16) $$\sum_{y=0}^{k^{2}-1}\sum_{z=0}^{k-1}\tau_{I}\left(\partial_{H}\left(E_{y}||F_{z}||G\right)\right)=r_{E_{\left\{a_{|k}\right\}\left\lfloor a\;/\;k\right\rfloor }}\left(d\right)\cdot s_{E_{\left\lfloor b\;/\;k\right\rfloor }\left\{b_{|k}\right\}}\left(e\right)\cdot \tau_{I}\left(\partial_{H}\left(E_{y}||F_{z}||G\right)\right)$$

On the other hand, here it comes the external behavior of the real system, where its incoming port is called IN and its outgoing port is named OUT, as seen in Equation (17):

(17) $$X=r_{IN}\left(d\right)\cdot s_{OUT}\left(e\right)\cdot X$$

By undertaking a comparison of both previous expressions, it may seem clear that both are recursive equations being multiplied by the same factors, so they obviously share the same string of actions and the same branching structure, leading to Equation (18):

(18) $$\sum_{y=0}^{k^{2}-1}\sum_{z=0}^{k-1}\tau_{I}\left(\partial_{H}\left(E_{y}||F_{z}||G\right)\right)⟷X$$

Hence, that is a sufficient condition to have a model verified. Therefore, the ACP model presented herein may be considered as duly verified.

### 6.2. Fog Full Mesh Scenario

To start with, there are only three entities involved in this scenario to be modeled, such as devices (*D*), edge nodes (*E*) and fog nodes (*F*), as there is no cloud node, whereas *p* and *q* denote generic source downlink ports within a given item. Moreover, the description of *D* and *E* matches those stated in the previous case, whilst the difference is in the behavior of *F*, where there is always a direct channel between a source fog $F_{\left\lfloor a\;/\;k^{2}\right\rfloor }$ and a destination fog $F_{\left\lfloor b\;/\;k^{2}\right\rfloor }$, with the source end of such a channel is located in the former and the destination end is situated in the latter. Moreover, the ${\mathrm{AI}}_{\mathrm{fog}}$ is applied in the common fog node in case of intrafog traffic flows, whilst it is applied in the destination fog node is case of interfog communications, that being cited as ${\mathrm{AI}}_{{\mathrm{fog}}^{\prime }}$, as it is exhibited in Equation (19).

(19) $$\begin{array}{c} F_{z}=\sum_{z=0}^{k-1}\sum_{q=0}^{k-1}(r_{\left\{q\right\}F_{z}}\left(d\right)\cdot (s_{F_{z}\left\{{\left\lfloor b\;/\;k\right\rfloor }_{|k}\right\}}\left(e\right)◃\;{\mathrm{AI}}_{\mathrm{fog}}\;▹\\ s_{F_{z}\left\{k+F_{\left\lfloor b\;/\;k^{2}\right\rfloor }-\left\lceil \frac{\left\lfloor F_{\left\lfloor b\;/\;k^{2}\right\rfloor }\;/\;(F_{\left\lfloor a\;/\;k^{2}\right\rfloor }+1)\right\rfloor }{k}\right\rceil \right\}}\left(d\right))+\\ r_{\left\{k+F_{\left\lfloor a\;/\;k^{2}\right\rfloor }-\left\lceil \frac{\left\lfloor F_{\left\lfloor a\;/\;k^{2}\right\rfloor }\;/\;(F_{\left\lfloor b\;/\;k^{2}\right\rfloor }+1)\right\rfloor }{k}\right\rceil \right\}F_{z}}\left(d\right)\cdot {\mathrm{AI}}_{{\mathrm{fog}}^{\prime }}\cdot s_{F_{z}\left\{{\left\lfloor b\;/\;k\right\rfloor }_{|k}\right\}}\left(e\right))\cdot F_{z} \end{array}$$

At this stage, the encapsulation operator may be applied so as to attain a sequence of events within the model of intermediate items, thus taking devices as external items, as indicated in Equation (20):

(20) $$\begin{array}{c} \sum_{y=0}^{k^{2}-1}\sum_{z=0}^{k-1}\partial_{H}\left(E_{y}||F_{z}||G\right)=(r_{\left\{a_{|k}\right\}E_{\left\lfloor a\;/\;k\right\rfloor }}\left(d\right)\cdot ({\mathrm{AI}}_{\mathrm{edge}}◃\;\left\lfloor a\;/\;k\right\rfloor =\left\lfloor b\;/\;k\right\rfloor \;▹\\ c_{E_{\left\lfloor a\;/\;k\right\rfloor }\left\{k\right\}\to \left\{{\left\lfloor a\;/\;k\right\rfloor }_{|k}\right\}F_{\left\lfloor a\;/\;k^{2}\right\rfloor }}\left(d\right)\cdot ({\mathrm{AI}}_{\mathrm{fog}}◃\;\left\lfloor a\;/\;k^{2}\right\rfloor =\left\lfloor b\;/\;k^{2}\right\rfloor \;▹\\ c_{F_{\left\lfloor a\;/\;k^{2}\right\rfloor }\left\{k+F_{\left\lfloor b\;/\;k^{2}\right\rfloor }-\left\lceil \frac{\left\lfloor F_{\left\lfloor b\;/\;k^{2}\right\rfloor }\;/\;(F_{\left\lfloor a\;/\;k^{2}\right\rfloor }+1)\right\rfloor }{k}\right\rceil \right\}\to \left\{k+F_{\left\lfloor a\;/\;k^{2}\right\rfloor }-\left\lceil \frac{\left\lfloor F_{\left\lfloor a\;/\;k^{2}\right\rfloor }\;/\;(F_{\left\lfloor b\;/\;k^{2}\right\rfloor }+1)\right\rfloor }{k}\right\rceil \right\}F_{\left\lfloor b/k^{2}\right\rfloor }}\left(d\right))\cdot \\ {\mathrm{AI}}_{{\mathrm{fog}}^{\prime }}\cdot c_{F_{\left\lfloor b/k^{2}\right\rfloor }\left\{{\left\lfloor b\;/\;k\right\rfloor }_{|k}\right\}\to \left\{k\right\}E_{\left\lfloor b\;/\;k\right\rfloor }}\left(e\right))\cdot s_{E_{\left\lfloor b\;/\;k\right\rfloor }\left\{b_{|k}\right\}}\left(e\right))\cdot \partial_{H}\left(E_{y}||F_{z}||G\right) \end{array}$$

It is to be noted that after the application of the abstraction operator, the results obtained are analogous to those achieved in the intracloud case scenario, as the external behavior matches in both cases.

### 6.3. Fog Hybrid Scenario

It is to be considered that this case is just a mixture of both previous cases, thus allowing for two different ways to face interfog communications, which is denoted by the + operator. Hence, the four entities are modeled, such as devices (*D*), edge nodes (*E*), fog nodes (*F*) and cloud node (*G*), where they are all like in the first case, except for the fog nodes (*F*), where intracloud and full mesh paths may be both available. In addition, it is to be noted that its port going towards the cloud is labeled as 2k−1, thus keeping the same uplink port scheme presented in the full mesh case scenario, as it is shown in Equation (21):

(21) $$\begin{array}{c} F_{z}=\sum_{z=0}^{k-1}\sum_{q=0}^{k-1}(r_{\left\{q\right\}F_{z}}\left(d\right)\cdot ({\mathrm{AI}}_{\mathrm{fog}}\cdot s_{F_{z}\left\{{\left\lfloor b\;/\;k\right\rfloor }_{|k}\right\}}\left(e\right)◃\;\left\lfloor a\;/\;k^{2}\right\rfloor =\left\lfloor b\;/\;k^{2}\right\rfloor \;▹\\ \left(s_{F_{z}\left\{2k-1\right\}}\left(d\right)+s_{F_{z}\left\{k+F_{\left\lfloor b\;/\;k^{2}\right\rfloor }-\left\lceil \frac{\left\lfloor F_{\left\lfloor b\;/\;k^{2}\right\rfloor }\;/\;(F_{\left\lfloor a\;/\;k^{2}\right\rfloor }+1)\right\rfloor }{k}\right\rceil \right\}}\left(d\right)\right))+\\ \left(r_{\left\{k\right\}F_{z}}\left(e\right)+r_{\left\{k+F_{\left\lfloor a\;/\;k^{2}\right\rfloor }-\left\lceil \frac{\left\lfloor F_{\left\lfloor a\;/\;k^{2}\right\rfloor }\;/\;(F_{\left\lfloor b\;/\;k^{2}\right\rfloor }+1)\right\rfloor }{k}\right\rceil \right\}F_{z}}\left(d\right)\cdot {\mathrm{AI}}_{{\mathrm{fog}}^{\prime }}\right)\cdot s_{F_{z}\left\{{\left\lfloor b\;/\;k\right\rfloor }_{|k}\right\}}\left(e\right))\cdot F_{z} \end{array}$$

At this stage, the encapsulation operator may be applied so as to achieve a sequence of events within the model of intermediate items, thus classifying devices as external items, as seen in Equation (22):

(22) $$\begin{array}{c} \sum_{y=0}^{k^{2}-1}\sum_{z=0}^{k-1}\partial_{H}\left(E_{y}||F_{z}||G\right)=(r_{\left\{a_{|k}\right\}E_{\left\lfloor a\;/\;k\right\rfloor }}\left(d\right)\cdot ({\mathrm{AI}}_{\mathrm{edge}}◃\;\left\lfloor a\;/\;k\right\rfloor =\left\lfloor b\;/\;k\right\rfloor \;▹\\ c_{E_{\left\lfloor a\;/\;k\right\rfloor }\left\{k\right\}\to \left\{{\left\lfloor a\;/\;k\right\rfloor }_{|k}\right\}F_{\left\lfloor a\;/\;k^{2}\right\rfloor }}\left(d\right)\cdot ({\mathrm{AI}}_{\mathrm{fog}}◃\;\left\lfloor a\;/\;k^{2}\right\rfloor =\left\lfloor b\;/\;k^{2}\right\rfloor \;▹\\ (c_{F_{\left\lfloor a\;/\;k^{2}\right\rfloor }\left\{k\right\}\to \left\{{\left\lfloor a\;/\;k^{2}\right\rfloor }_{|k}\right\}G_{\left\lfloor a\;/\;k^{3}\right\rfloor }}\left(d\right)\cdot {\mathrm{AI}}_{\mathrm{cloud}}\cdot c_{G_{\left\lfloor b\;/\;k^{3}\right\rfloor }\left\{{\left\lfloor b\;/\;k^{2}\right\rfloor }_{|k}\right\}\left\{k\right\}F_{\left\lfloor b\;/\;k^{2}\right\rfloor }}\left(e\right)\;+\\ c_{F_{\left\lfloor a\;/\;k^{2}\right\rfloor }\left\{k+F_{\left\lfloor b\;/\;k^{2}\right\rfloor }-\left\lceil \frac{\left\lfloor F_{\left\lfloor b\;/\;k^{2}\right\rfloor }\;/\;(F_{\left\lfloor a\;/\;k^{2}\right\rfloor }+1)\right\rfloor }{k}\right\rceil \right\}\to \left\{k+F_{\left\lfloor a\;/\;k^{2}\right\rfloor }-\left\lceil \frac{\left\lfloor F_{\left\lfloor a\;/\;k^{2}\right\rfloor }\;/\;(F_{\left\lfloor b\;/\;k^{2}\right\rfloor }+1)\right\rfloor }{k}\right\rceil \right\}F_{\left\lfloor b\;/\;k^{2}\right\rfloor }}\left(d\right)\cdot \\ {\mathrm{AI}}_{{\mathrm{fog}}^{\prime }}))\cdot c_{F_{\left\lfloor b\;/\;k^{2}\right\rfloor }\left\{{\left\lfloor b\;/\;k\right\rfloor }_{|k}\right\}\to \left\{k\right\}E_{\left\lfloor b\;/\;k\right\rfloor }}\left(e\right))\cdot s_{E_{\left\lfloor b\;/\;k\right\rfloor }\left\{b_{|k}\right\}}\left(e\right))\cdot \partial_{H}\left(E_{y}||F_{z}||G\right) \end{array}$$

As stated in the fog full mesh case scenario, it is to be noted that after applying the abstraction operator, the results attained are analogous to those obtained in the intracloud case scenario, as the external behavior matches in both cases.

## 7. Conclusions

In this paper, an arithmetic framework for numbering nodes and devices, along with their ports, aimed at generic edge computing environments has been proposed. To start with, a small introduction on edge computing has been developed, followed by some mathematical background regarding integer divisions and modular arithmetic. Afterwards, the foundation of the edge computing model proposed has been exposed by presenting the different layers being part of it, along with the representation of items and their ports located on each of those layers.

At that stage, the features of the MEC model proposed have been exhibited, such as intraedge and intrafog communications, followed by three different approaches related to interfog one. After that, diverse theorems have been presented so as to assure that each of those communications do really take place with the MEC model proposed. Eventually, all three diverse scenarios have been modeled with ACP in order to find out whether the external behavior of such models match those of the real system being modeled, concluding that all models become duly verified.

## Figures and Tables

**Table 1:** Alternative ways for calculating the quotient when dealing with integer division.

Type of Integer Division	Characteristic Feature
Standard Euclidean division	Remainder is always positive
Floored division	Remainder always has the same sign of the divisor
Ceiled division	Remainder always has the opposite sign of the divisor
Rounded division	Remainder always has the least absolute value
Truncated division	Remainder always has the same sign as the Dividend
Negative remainders	Remainder is always negative
Greatest absolute value	Remainder always has the greatest absolute value
Different sign from dividend	Remainder always has the different sign from the Dividend

**Table 2:** Number of devices for each layer in a hub and spoke interfog scenario within the MEC framework proposed.

*Layer*	*Items* *in Any Layer*	*Items* *per Edge Layer*	*Items* *per Fog Layer*	*Items* *per Cloud Layer*
*Cloud*	$k^{0}=\;$ *1*	*N/A*	*N/A*	*1*
*Fog*	$k^{1}=k$	*N/A*	*1*	*k*
*Edge*	$k^{2}$	*1*	*k*	$k^{2}$
*IoT Devices*	$k^{3}$	*k*	$k^{2}$	$k^{3}$

**Table 3:** Number of devices for each layer in a full mesh interfog scenario within the MEC framework proposed.

*Layer*	*Items* *in Any Layer*	*Items* *per Edge Layer*	*Items* *per Fog Layer*	*Items* *per Cloud Layer*
*Cloud*	$k^{0}=1$	*N/A*	*N/A*	*N/A*
*Fog*	$k^{1}=k$	*N/A*	*1*	*k*
*Edge*	$k^{2}$	*1*	*k*	$k^{2}$
*IoT Devices*	$k^{3}$	*k*	$k^{2}$	$k^{3}$

**Table 4:** Channels defined within the model and their nomenclature.

Channel Name			Nomenclature		
source device	→	source edge	$D_{a}\left\{0\right\}$	→	$\left\{a_{|k}\right\}E_{\left\lfloor a/k\right\rfloor }$
source edge	→	source fog	$E_{\left\lfloor a/k\right\rfloor }\left\{k\right\}$	→	$\left\{{\left\lfloor a/k\right\rfloor }_{|k}\right\}F_{\left\lfloor a/k^{2}\right\rfloor }$
source fog	→	cloud	$F_{\left\lfloor a/k^{2}\right\rfloor }\left\{k\right\}$	→	$\left\{{\left\lfloor a/k^{2}\right\rfloor }_{|k}\right\}G_{\left\lfloor a/k^{3}\right\rfloor }$
cloud	→	dest.fog	$G_{\left\lfloor b/k^{3}\right\rfloor }\left\{{\left\lfloor b/k^{2}\right\rfloor }_{|k}\right\}$	→	$\left\{k\right\}F_{\left\lfloor b/k^{2}\right\rfloor }$
dest.fog	→	dest.edge	$F_{\left\lfloor b/k^{2}\right\rfloor }\left\{{\left\lfloor b/k\right\rfloor }_{|k}\right\}$	→	$\left\{k\right\}E_{\left\lfloor b/k\right\rfloor }$
dest.edge	→	dest.device	$E_{\left\lfloor b/k\right\rfloor }\left\{b_{|k}\right\}$	→	$\left\{0\right\}D_{b}$
source fog ($F_{i}$)	→	dest.fog ($F_{j}$)	$F_{i}\left\{k+F_{j}-\left\lceil \frac{\left\lfloor F_{j}\;/\;\left(F_{i}+1\right)\right\rfloor }{k}\right\rceil \right\}$	→	$\left\{k+F_{i}-\left\lceil \frac{\left\lfloor F_{i}\;/\;\left(F_{j}+1\right)\right\rfloor }{k}\right\rceil \right\}F_{j}$

**Table 5:** Channels defined within the model and their usages.

Channel Name			Model Cloud	Model Full Mesh	Model Hybrid
source device	→	source edge	✓	✓	✓
source edge	→	source fog	✓	✓	✓
source fog	→	cloud	✓		✓
cloud	→	dest. fog	✓		✓
dest. fog	→	dest. edge	✓	✓	✓
dest. edge	→	dest. device	✓	✓	✓
source fog ($F_{i}$)	→	dest. fog ($F_{j}$)		✓	✓

## Data Availability

Not applicable.

## Abbreviations

The following abbreviations are used in this manuscript:

ACP	Algebra of Communicating Processes
AI	Artificial Intelligence
CNN	Convolutional Neural Networks
DS	Data Science
FDT	Formal Description Techniques
IoT	Internet of Things
LAN	Local Area Network
MEC	Multi-Access Edge Computing
OSI	Open Systems Interconnection
WAN	Wide Area Network
